# Editorial: The Impact of Adipose Tissue Dysfunction on Cardiovascular and Renal Disease

**DOI:** 10.3389/fendo.2021.815894

**Published:** 2022-01-04

**Authors:** Xiaodong Sun, Cheng-Chao Ruan, Alexandre A. da Silva

**Affiliations:** ^1^ Department of Endocrinology and Metabolism, Affiliated Hospital of Weifang Medical University, Weifang, China; ^2^ Branch of Shandong Provincial Clinical Research Center for Diabetes and Metabolic Diseases, Affiliated Hospital of Weifang Medical University, Weifang, China; ^3^ Clinical Research Center, Affiliated Hospital of Weifang Medical University, Weifang, China; ^4^ Shanghai Key Laboratory of Bioactive Small Molecules, Department of Physiology and Pathophysiology, School of Basic Medical Sciences, Fudan University, Shanghai, China; ^5^ Department of Physiology and Biophysics, Mississippi Center for Obesity Research, and Cardiorenal and Metabolic Diseases Research Center, University of Mississippi Medical Center, Jackson, MS, United States

**Keywords:** abdominal aortic aneurysm (AAA), PVAT, perivascular adipose tissue, obesity, vascular, vascular diseases, adipose tissue

Obesity is considered an adverse metabolic disease with high risk of vascular complications, including cardiovascular disease (CVD) and chronic kidney disease (CKD). These obesity-related complications are strongly related to excess adipose tissue accumulation, with earlier studies centered on the impact of typical visceral versus subcutaneous adipose tissue accumulation. However, the adipose tissues around blood vessels and organs, including perivascular adipose tissue (PVAT), pericardial adipose tissue (PAT), epicardial adipose tissue (EAT) and perirenal adipose tissue (PRAT), have attracted a great deal of attention recently. Dysfunction in these “novel” adipose tissues have been associated with greater risk of CVD and CKD in obesity than in traditional visceral adipose tissue. Therefore, studies on the interactions of various adipose tissue malfunctions to metabolic-related diseases are vital for improved prevention and treatment of cardiometabolic and renal diseases. This Research Topic aimed to provide a platform for novel advances in dysfunction of various adipose tissues to identify significant contributors to metabolism-related vascular complications, especially CVD and CKD. The Research Topic represents a collection of 7 review articles and 3 original research articles ranging from basic studies to clinical trials, which provide insights into better understanding of the pathophysiology of adipose dysfunction in CVD and CKD.

PVAT, a fat pad surrounding the blood vessels, maintains normal vascular function. However, PVAT becomes dysfunctional in obesity and other pathological conditions. The review by Chen et al. summarizes recent findings regarding dysfunction and inflammation of PVAT in vascular activity, vascular aging, hypertension, atherosclerosis, diabetes and a few other pathological conditions. This review article highlights how PVAT malfunction promotes vascular diseases through stimulation of inflammatory factors and various secreted adipokines, and that anti-inflammatory therapy to mitigate PVAT inflammation may be a valuable target for future treatment of vascular diseases.

Abdominal aortic aneurysms (AAAs) are irreversible vascular diseases with high mortality rates associated with aneurysm rupture. The association between PVAT and AAA development was reviewed by Ye et al. Factors derived from PVAT are involved in all stages of AAA disease development, including inflammatory cell infiltration, the onset of oxidative stress, and matrix metalloproteinase activation. Excessive adipocyte accumulation derived from PVAT in ruptured AAA walls is closely linked with AAA progression. Through discussion of what is known about PVAT-derived factors, this review highlights the need for additional studies into identifying therapeutic drugs targeted for PVAT dysfunction to reduce AAA rupture-induced mortality.

PVAT dysfunction following vascular damage also promotes neointimal formation by releasing adipocytokines that can regulate the phenotypic switch of vascular smooth muscle cells. Original research studies by Lei et al. found that MIA3/TANGO1 regulates vascular remodeling response to injury. MIA3/TANGO1 deficiency inhibits neointimal formation by preventing vascular smooth muscle cell proliferation/migration, ameliorating neointimal hyperplasia, and maintaining PVAT function during injury-induced vascular remodeling. MIA3/TANGO1 may be a novel potential target for neointimal formation after vascular injury.

Given the interactions between adipose and CVD, several authors centered their work on the cross-talk between the adipose tissue surrounding the heart or myocardium (PAT or EAT) and coronary artery disease (CAD). Using a variety of bioinformatics algorithms, Li et al. highlighted potential roles of molecular alterations in PAT and its association with CAD. 147 differently expressed genes and altered predicted pathways were identified to be mainly associated with regulation of immune system and inflammatory response in PAT of patients with CAD. With the outbreak of coronavirus disease 2019 (COVID-19), patients with metabolic disease and high adverse cardiovascular events attracted attention. The review by Lasbleiza et al. emphasized the harmful role of excess EAT on myocardial injury among COVID-19 patients with obesity. This inflamed EAT depot may participate in COVID-19-related cardiac injury due to its unique anatomical contact with the myocardium and its inflammatory status. The impact of adipose tissue dysfunction on CVD was also reviewed by Bermúdez et al. This review discussed how “sick” adipose tissue affects cardiac pathology, such as arterial fibrillation, coronary artery disease, and myocardial infarction. Changes in adipose tissue microenvironment and metabolic reprogramming in adipose tissue were also summarized in this article.

PRAT, a fat pad surrounding the kidney, has recently been implicated in the regulation of kidney function. In a Mini-Review, Hammoud et al. presented a general overview of new insights linking CVD and CKD, focusing on metabolic disturbances affecting the physiological function of PRAT and potential mechanisms. The review summarized PRAT regulation in various metabolic states and CKD and strengthened the vital role of PRAT, a usually neglected adipose tissue, on regulating homeostasis.

The gut microbiome has also emerged as a critical regulator of host metabolism. It possesses specific impacts on systemic metabolisms and CVD. Yang et al. reviewed the role of gut microbiota and its metabolites in the development and pathogenesis of CVD caused by adipose tissue dysfunction and potential targeted therapies against undesirable gut microbiota. The authors also summarized the present state of clinical therapies for adipose tissue dysfunction targeting the gut microbiota.

Sestrin2, a highly conserved stress-induced protein, may represent a novel antioxidant target for metabolic diseases. Gong et al. include a balanced and comprehensive view of specific mechanisms of Sestrin2 actions in the development of different diseases. This review briefly introduced the potential for Sestrin2 as a clinical marker or therapeutic target. It primarily summarized the regulatory interactions between Sestrin2 and AMPK/mTOR signaling and the effects of Sestrin2 on glucose and lipid metabolism, aging and myocardial energy metabolism.

Telomere shortening and telomerase activity caused by inflammation and oxidative stress are also associated with cardiovascular risk. Li et al. investigated the telomerase RNA component (TERC), telomerase reverse transcriptase (TERT) gene variants, and acute heart failure (AHF) in a prospective study that enrolled 322 patients. The authors found that seven single nucleotide polymorphisms (SNPs) of TERC and TERT are independent risk factors for predicting 18-month mortality in AHF.

Overall, the articles outlined above in this Research Topic underscore the critical role of various adipose tissues dysfunction in CVD and CKD progression, and poses a timely question of whether targeting specific adipose tissue depots may become an important target for better, more efficacious approaches to prevent and/or treat CVD and CKD.

## Author Contributions

All authors contributed to the article and approved it for publication.

## Funding

This work was supported by the National Natural Science Foundation of China (81870593, 82170865).

## Conflict of Interest

The authors declare that the research was conducted in the absence of any commercial or financial relationships that could be construed as a potential conflict of interest.

## Publisher’s Note

All claims expressed in this article are solely those of the authors and do not necessarily represent those of their affiliated organizations, or those of the publisher, the editors and the reviewers. Any product that may be evaluated in this article, or claim that may be made by its manufacturer, is not guaranteed or endorsed by the publisher.

